# Circulating microRNAs as predictors of response to sofosbuvir + daclatasvir + ribavirin in in HCV genotype-4 Egyptian patients

**DOI:** 10.1186/s12876-022-02485-6

**Published:** 2022-12-03

**Authors:** Noha Anwar Hassuna, Aya Nabil Gamil, Mahmoud Shokry Mahmoud, Wafaa Khairy Mohamed, Rasha Khairy

**Affiliations:** Department of Medical Microbiology and Immunology, Faculty of Medicine, Minia University, Minia, Egypt

**Keywords:** MicroRNAs, HCV (genotype-4), Sofosbuvir, Daclatasvir, Liver fibrosis

## Abstract

**Background:**

MicroRNAs (miRNAs) play an important role in various diseases, including HCV infection, the aim of the current study was to evaluate the potential use of serum miRNAs as biomarkers for diagnosis, prognosis, and prediction of responses to direct acting antivirals (sofosbuvir + daclatasvir + ribavirin) in HCV-4 patients.

**Methods:**

The serum expression profiles of four liver-associated miRNAs (miRNA‐122, 155, 196 and 29) were assessed in 160 HCV-4 patients and 50 healthy controls using real-time PCR prior to therapy.

**Results:**

miR-122 and miR-155 showed upregulation in HCV-4 patients compared to healthy controls while miR-196 and miR-29 showed downregulation in HCV-4 patients. ROC curve analyses revealed that the four-studied miRNAs could be valuable biomarkers for predicting response to DAAs with AUC 0.973 for miR-122, 0.878 for miR-155, 0.808 for miR-29 and 0.874 for miR-196 respectively. Univariate logistic regression analysis revealed that miR-196 level is positive predictor for SVR, whereas miR-122,155 levels are negative predictors of response. Multivariate logistic regression analysis revealed that miR-196 is the most significant in predicting response to treatment (*p* value = 0.011).

**Conclusion:**

To the best of our knowledge, the current study provided the first clinical evidence of the potential use of circulating miRNAs (miR; 122, 155, 196 and 29) as biomarkers of CHC in HCV-4 patients receiving the new DAA regimen (SOF/DAV + RIB), which is a strong motivator for further studies.

## Background

With about 71 million people worldwide infected, hepatitis C virus (HCV) infection is a global health issue. It is a significant cause of chronic hepatitis, hepatic cirrhosis, hepatic cell carcinoma [1]. Unfortunately, Egypt has one of the highest  prevalence rates of HCV, worldwide; with approximately 14.7% of anti‐HCV antibodies [2]. The most predominant genotype in Egypt is HCV-4, which accounts for about 94.1% of infections [3].

Advances in anti-HCV therapy have jumped into a new era with interferon (IFN)—free regimens [4]**.** Discovery of direct acting antiviral (DAA) drugs that target non-structural proteins of HCV leading to termination of viral replication has dramatically increased the probability of viral eradication, with more than 90% success rate with respect to IFN-based regimens [5].

MiRNAs play an important role in directly regulating signaling pathways in the pathogenesis of HCV infection as they have a role in native and acquired immunity [6]. Furthermore, the high stability of miRNAs in serum and their sensitive detection by quantitative PCR result in their potential value as non-invasive diagnostic and prognostic biomarkers for liver disease in HCV infection. In addition, circulatory miRNAs are emerging as a tool to detect therapeutic outcome and as therapeutic targets in HCV infection [7].

MiR-122 is considered the most abundant miRNA in normal liver parenchyma, accounting for over 70% of the total miRNAs in hepatocytes [8]. MiR-122 binds to the 5′-UTR of HCV RNA and this is critical for viral replication, it stimulates viral protein translation and protects the uncapped HCV RNA from degradation.

The observation of MiR-155 upregulation in the serum, PBMCs, and hepatocytes of HCV-infected individuals points to a potential involvement for this gene in the aetiology and development of chronic HCV infection and HCC-related HCV [9]. The degree of miR-155 expression may be helpful in determining how effective a therapy is; if miR-155 is overexpressed, viral persistence in PBMCs and poor treatment effectiveness may be foreseen. The use of MiR-155 antagonists in the treatment of chronic HCV infections might prevent additional hepatocellular damage from occurring.

In contrast to miR-155, miR-196b suppresses viral replication by attaching to the NS5A region and promotes the production of heme oxygenase 1 (HMOX1), which possesses cytoprotective, anti-inflammatory, and antioxidant properties. As a result, miR-196b mimics might be applied to the treatment of HCV to boost its effectiveness and prevent or even reverse liver damage. A possible favourable prognostic and predictive biomarker for HCV infection may be MiR-196b overexpression in the serum of affected individuals [10].

miR-29a is reported to have anti-viral and anti-fibrotic effects. It was shown to decease the HCV replication [11].

The aim of the current study was to evaluate the potential use of serum miRNAs as biomarkers for diagnosis, prognosis and prediction of responses to direct acting antivirals (sofosbuvir + daclatasvir + ribavirin) in HCV-4 patients.

## Patients and methods

### Study design

This study was carried out in Microbiology and Immunology department, Faculty of Medicine, Minia University, between February 2017 and December 2019.

Reverse transcriptase polymerase chain reaction (RT-PCR) was used to detect HCV RNA in 160 chronic HCV-4 patients who had visited the outpatient clinics of the Minia Health Insurance facility and tested positive for anti-HCV antibodies by ELISA (a referral centre for treatment of HCV-patients in Egypt). Fifty healthy, age- and gender-matched volunteers without a history of sickness and negative for hepatitis virus infection were assessed as controls. According to the "Declaration of Helsinki" principles and with the approval of Minia University's Research Ethics Committee (REC), written informed permission was acquired from patients and controls before to their involvement in the study. Patients with hepatocellular carcinoma, autoimmune hepatitis, alcohol-induced liver damage, the hepatitis B virus, HIV infection, active schistosomiasis, renal insufficiency, clinically overt diabetes mellitus, thyroid dysfunction, or any other endocrine, haematological, or chronic disease were excluded.

### Treatment regimen

All patients followed the new direct-acting antiviral regimen which consisted of combination of Daclatasvir (1 tablet; 60 mg/day), Sofosbuvir (1 tablet; 400 mg/ day) + Ribavirin (800 mg/day) (SOF/DAV + RIB) for 12 weeks. All patients including responders and non-responders were DAA treatment naive.

### Laboratory investigations and clinical assays

Each individual had a sample of 10 ml of venous blood drawn that was taken under strict aseptic conditions. The supernatant serum from one portion—which had been allowed to clot—was centrifuged and utilised for laboratory tests on the complete blood count (CBC), aspartate transferase (AST), and albumin levels. The enzyme-linked immunosorbent test was used to measure antibodies for HCV, HBV, and HIV (ELISA). Using quantitative real-time PCR (Qiagen), serum HCV RNA levels were assessed before to and six months after the conclusion of therapy. The tests were all conducted in accordance with the manufacturer's instructions. Before starting treatment, abdomen ultrasonography was performed on all HCV patients to determine the degree of fibrosis.

### Patients’ grouping

Based on the final virological response to DAAs/RBV therapy, all patients were categorized after completing the treatment plan for a total of 12 weeks. The study included a total of 150 HCV-4 patients, of which 141 achieved SVR, which was defined as negative HCV RNA by PCR six months after the end of treatment, plus 9 non-responders (NR), who did not achieve a negative HCV RNA by PCR. Patients who voluntarily discontinued therapy or dropped out were excluded from the study.

### Serum miRNAs assay: RNA extraction

Total RNA containing miRNAs was extracted using miRNeasy reaction kit (Qiagen, USA) from 200 μl pretreatment serum samples according to manufacturer’s instructions. Nanodrop was used to determine RNA quality (Thermo scientific, USA).

### Reverse transcription (RT)

The reaction was carried out on 100 ng of total RNA in a final volume of 20 μl RT reactions using the miRNeasy Reverse Transcription kit (Qiagen, USA) according to the manufacturer’s instructions.

### Quantitative real-time PCR

Real‐time PCR was used for relative quantification of four miRNAs (namely, miR‐122, miR‐155, miR‐196b, miR‐29) using miScript SYBR Green Master Mix reagents (Qiagen, USA) according to the manufacturer’s instructions. The miRNAs primers were supplied by Qiagen. Endogenous control was included using SNORD68. The reaction mixture was prepared with final volume of 25 μl as follows; 12.5 μl of QuantiTect SYBR Green PCR master mix, 2.5 μl of miScript Universal Primer, 2.5 μl of miScript primer assay, 5 μl of RNase-free water, 2.5 μl of diluted template cDNA. The cycling conditions were as follows: initial denaturation at 95 °C for 15 min, then 40 cycles at 94 °C for 15 s, 55 °C for 30 s, and 72 °C for 30 s. dissociation curve analysis of the PCR products was performed to verify their specificity.

### Relative quantification of miRNAs

The relative quantification (RQ) of the four miRNAs was analyzed using the comparative Ct method which was calculated by the following formulas:

- (1) ∆Ct = Ct (gene of interest) − Ct (housekeeping gene).
- (2) ∆∆Ct = ∆Ct (Sample)  − ∆Ct (Control average).
- (3) Relative quantification (RQ) = 2^−∆∆Ct.

### Statistical analysis

Statistical Package for Social Sciences (IBM SPSS Statistics for Windows, version XX (IBM Corp., Armonk, N.Y., USA) application version 24 was used to tabulate and statistically analyse the gathered data. When applicable, descriptive statistics were run on parametric quantitative data using the mean, standard deviation, median (25th–75th percentiles), or number (percentage). Using the Mann–Whitney U-test or the Student's t test, independent samples from two groups were compared. Serum miRNA expression levels and viral load were compared using the Mann–Whitney U-test since the data were not normally distributed.

Using the Chi squared (X2) test, categorical data were compared. The cut-off point, sensitivity, specificity, PPV, and NPV were computed using the receiver operating characteristic (ROC) curve and the area under the curve (AUC). To find factors that may predict how well a therapy will work, logistic regression analysis was used. Multivariate analysis was used to identify the independent factors that impacted the answer for data that were significant according to univariate analysis. Using Pearson's correlation coefficient, two quantitative variables were correlated. Weak (r = 0–0.24), fair (r = 0.25–0.49), moderate (r = 0.5–0.74), and strong (r = 0.75–1) were the correlation coefficient ranges from (0–1). (*p* value 0.05) was used to determine the significance threshold. Excel office 10 was used to do the calculations.

## Results

### Expression pattern of mirRNAs in HCV cases and controls

**Fig. 1:**
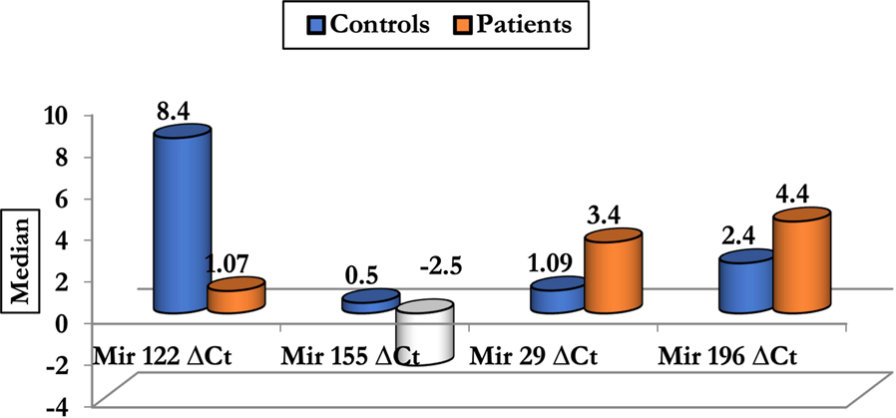
Expression pattern of mirRNAs in HCV cases (before treatment) and controls: Data are expressed as mean, SD, median (25–75% percentiles) and were analyzed by Mann–Whitney U test. The data was statistically significant (*p* < 0.001)

The current study reported a significant difference between patients and controls regarding miRNAs level where the levels of serum miR-122 and miR-155 were significantly higher in the chronic hepatitis C (CHC) patients than in healthy controls, On the other hand, the levels of serum miR-196 and miR-29 were significantly lower in the chronic hepatitis C patients than in healthy controls (Fig. 1).

### Correlation between miRNAs expression and viral load

**Fig. 2:**
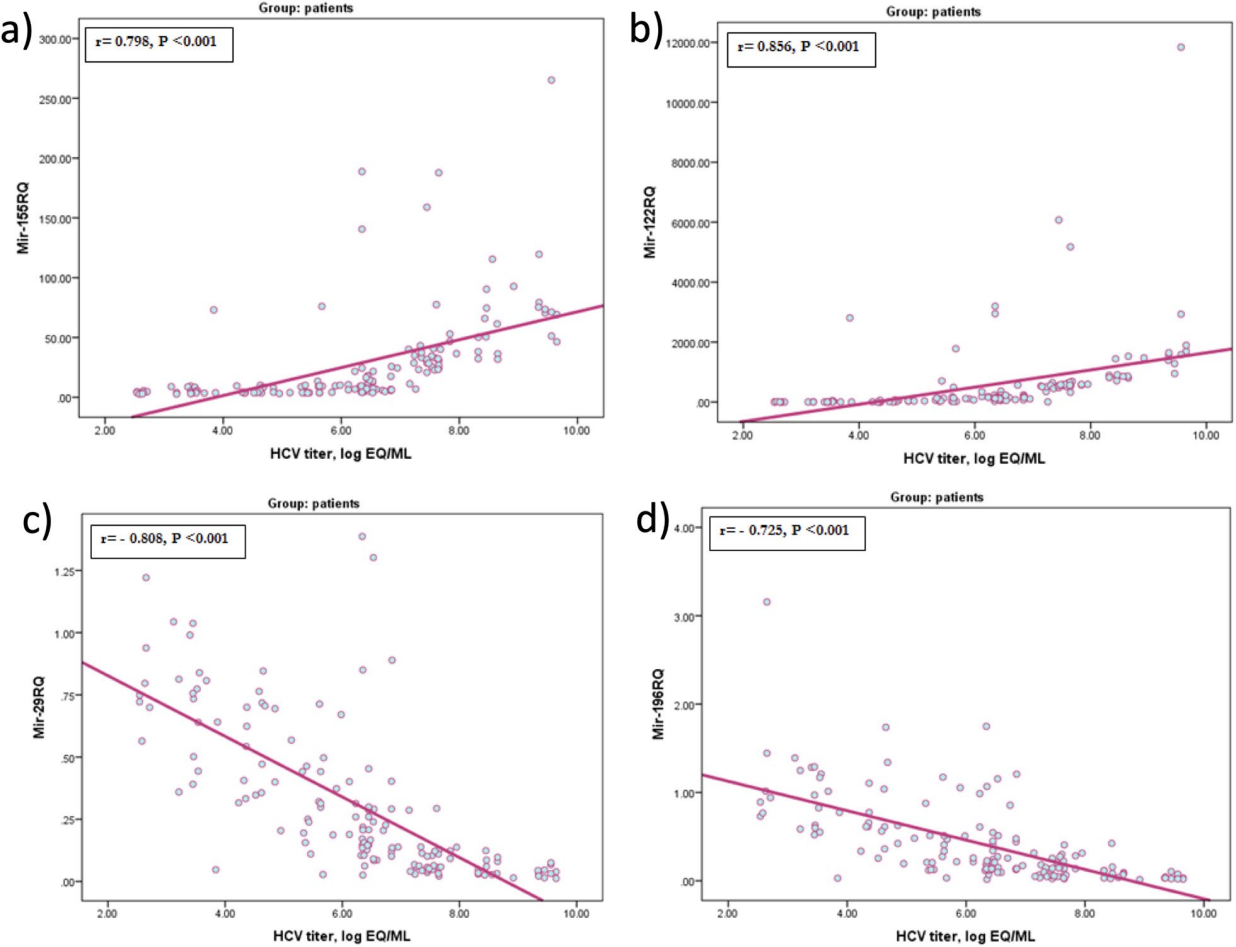
Correlation between miRNAs expression and viral load: **a** miRNA 155, **b** miRNA 122, **c** miRNA 29, **d** miRNA 196

Bivariate correlation analysis revealed positive significant strong correlation between mirRNAs 122, 155 and HCV RNA levels (r = 0.856, *p* < 0.001), (r = 0.798, *p* < 0.001), respectively. In contrast, it revealed negative significant strong and moderate correlation between mirRNAs 29, 196 and HCV RNA levels (r = − 0.808, *p* < 0.001), (r = − 0.725, *p* < 0.001), respectively (Fig. 2).

### Expression levels of miRNA in SVR and NR

**Fig. 3:**
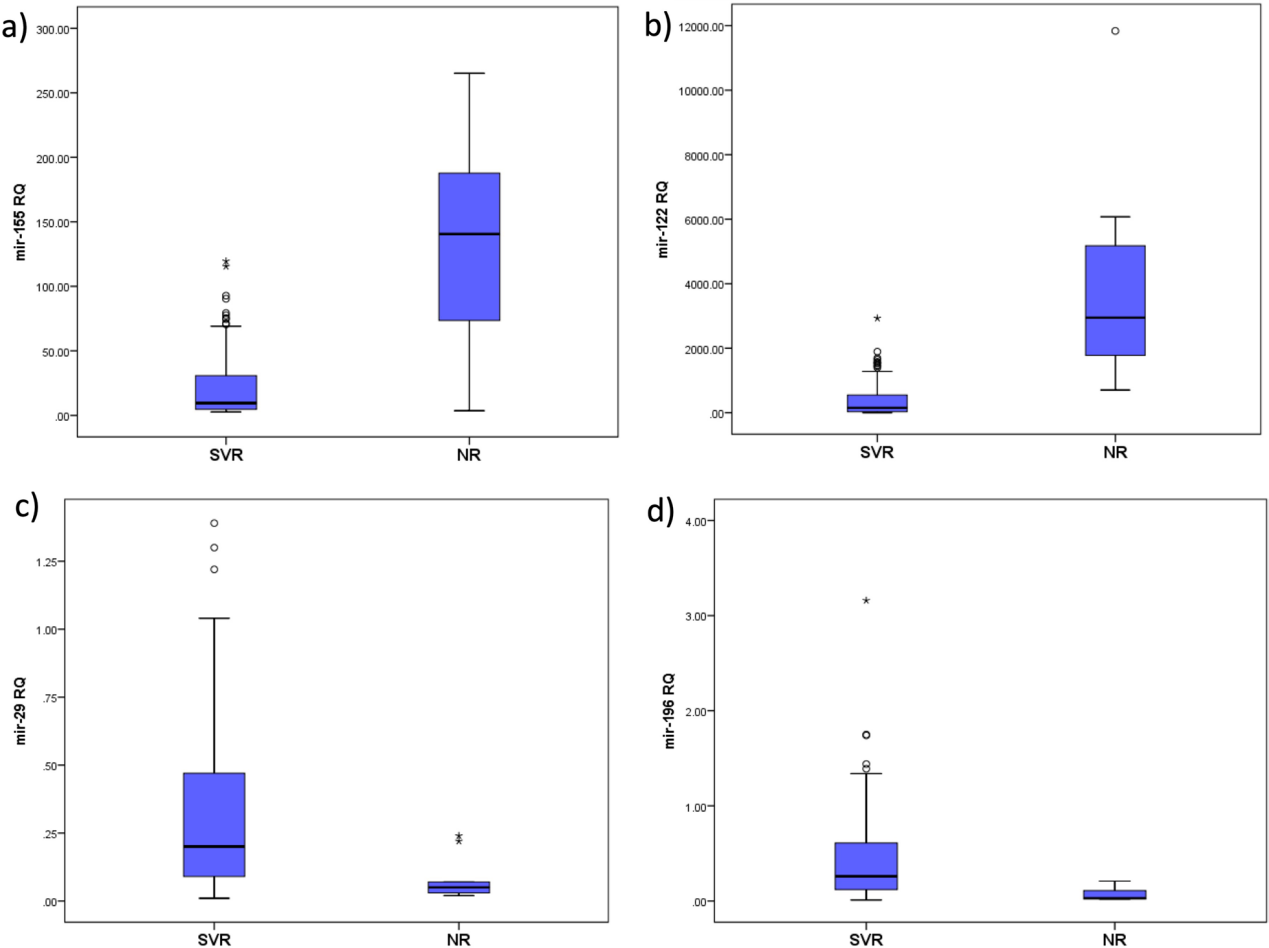
Serum miRNA expression levels in SVR and NR: **a** miRNA 155, **b** miRNA 122, **c** miRNA 29, **d** miRNA 196. The box represents the 25–75% percentiles; the line inside the box represents the median and the upper and lower lines representing the 10–90% percentiles of pretreatment expression levels of **a** miR-122, **b** miR-155, **c** miR-29, **d** miR-29 in SVR (n = 151) and NR (n = 9). Data were analyzed by Mann–Whitney U test. The data was statistically significant (*p* < 0.001)

The expression levels of miR-122 and miR-155 were significantly higher in non-responders to DAAs than in responders. In contrast, the expression levels of miR-29 and miR-196 were significantly higher in responders to DAAs than in non-responders (Fig. 3).

### ROC curve analysis of serum miRNAs as diagnostic biomarkers differentiating HCV patients from healthy controls

**Fig. 4:**
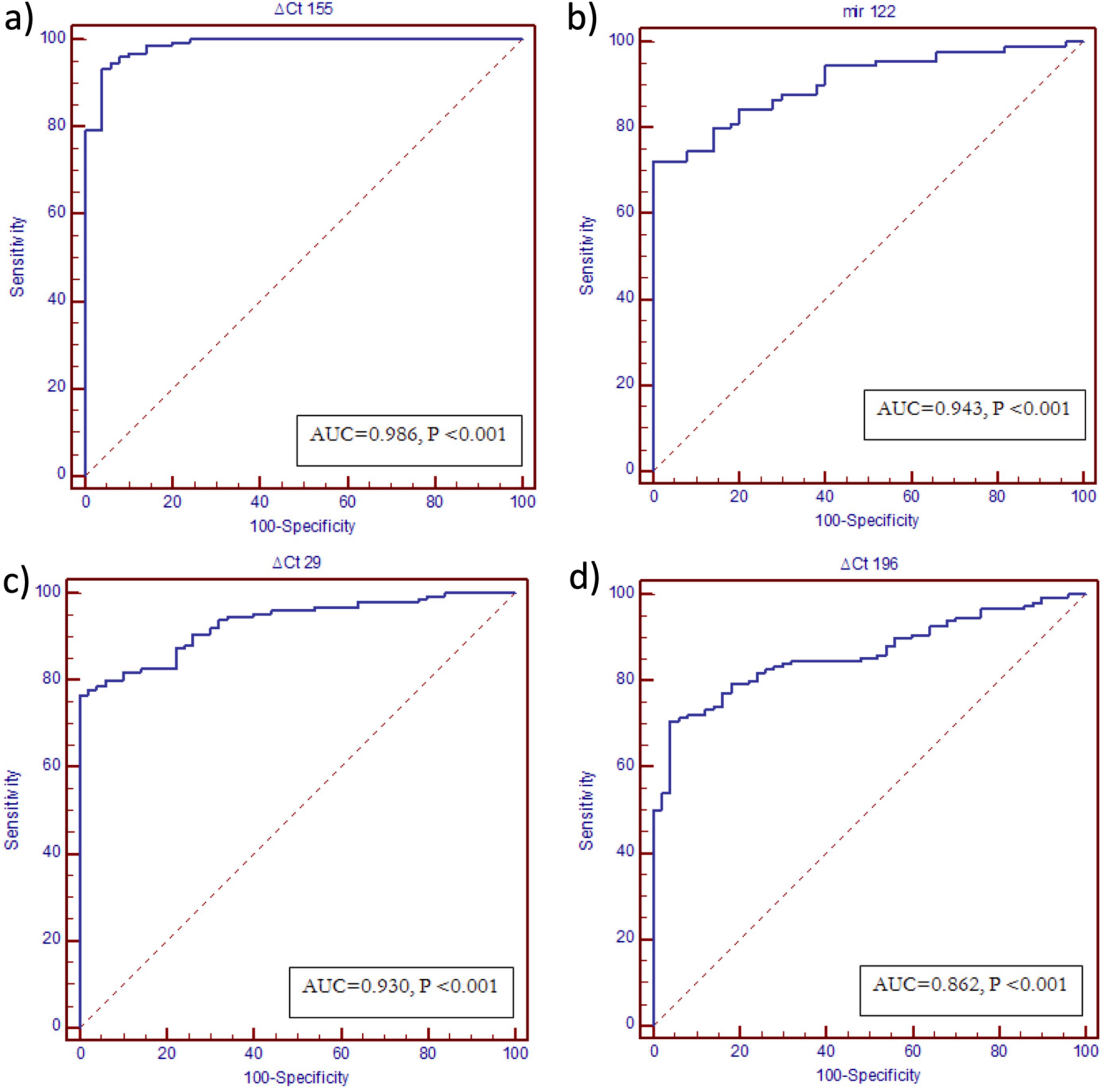
ROC curve analysis of serum miRNAs as diagnostic biomarkers differentiating HCV patients from healthy controls: **a** miRNA 155, **b** miRNA 122, **c** miRNA 29, **d** miRNA 196

ROC curve analysis was carried out to analyze the diagnostic accuracy of serum miRNAs. All the studied miRNAs could differentiate CHC patients from healthy controls with an AUC of 0.943 for miR-122 (95% CI 0.92–0.97, *p* < 0.001),0.986 for miR-155 (95% CI 0.958–0.997, *p*  < 0.001), 0.93 for miR-29 (95% CI 0.886–0.962, *p* < 0.001) and 0.862 for miR-196 (95% CI 0.66–0.86, *p* < 0.0001), respectively (Fig. 4). The optimal sensitivity and specificity to differentiate CHC patients from healthy controls were (83.3 and 100% at a cut-off value ≤ 4.6) for miR-122, (93.3 and 96% at a cut-off value ≤ − 1.11) for miR-155, (85.8 and 80.0% at a cut-off expression value > 1.01) for miR-29, (70.7and 96% at a cut-off value > 3.2) for miR-196, respectively. Comparison of the ROC curve results showed that the diagnostic accuracy of serum miR-155 was the most superior; in the order of miR-155 > miR-122 > miR-29 > miR 196 (AUC = 0.98, 0.94, 0.93 and 0.86, respectively) (Fig. 4).

### ROC curve analysis of serum miRNAs as predictive biomarker differentiating SVR and NR

**Fig. 5:**
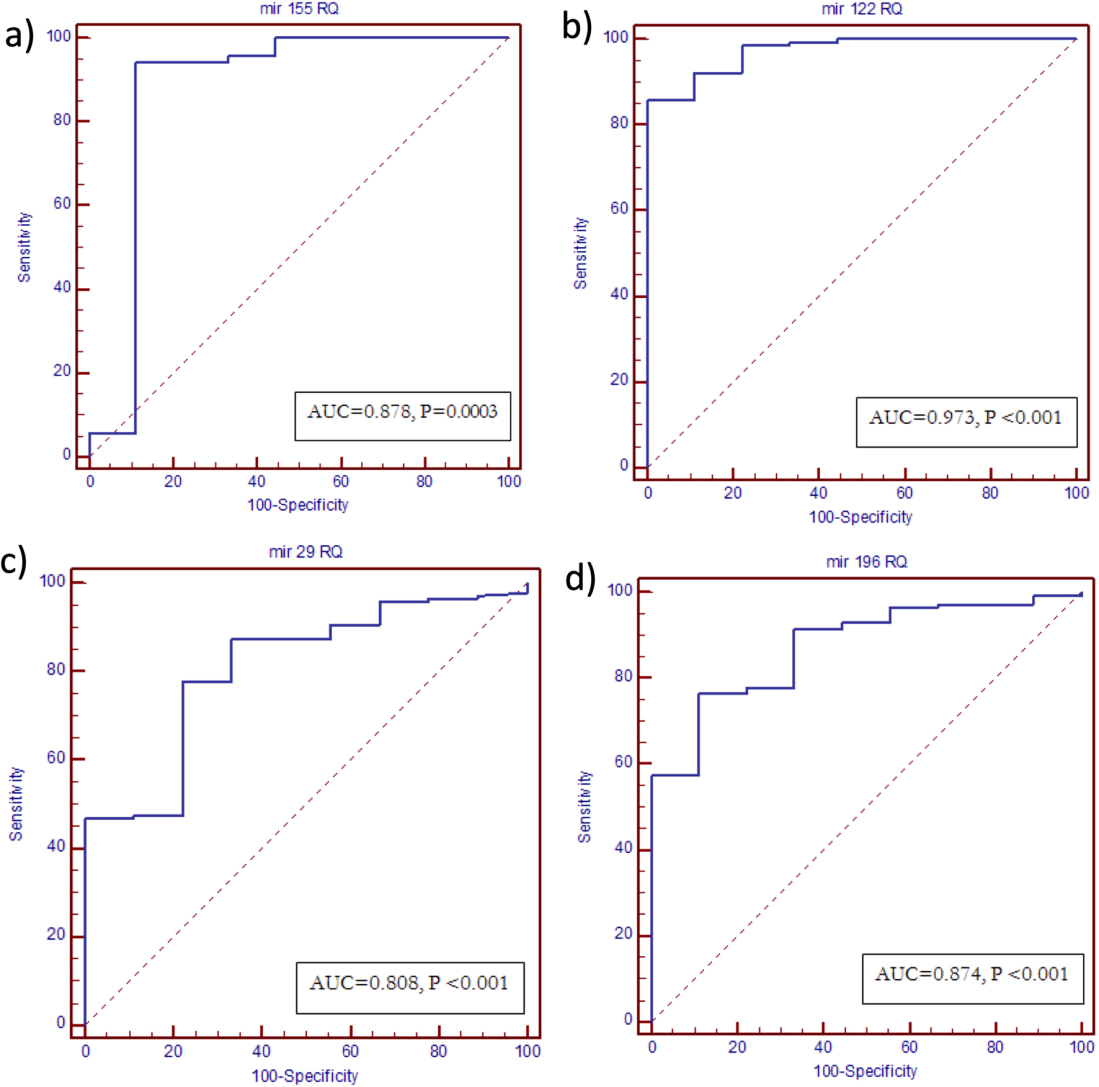
ROC curve analysis of serum miRNAs as predictive biomarker differentiating SVR and NR: **a** miRNA 155, **b** miRNA 122, **c** miRNA 29, **d** miRNA 196

All studied miRNAs could differentiate SVR from NR with an AUC of 0.973 for miR-122 (95% CI 0.933–0.993, *p* < 0.0001), 0.878 for miR-155 (95% CI 0.815–0.926, *p* = 0.0003), 0.808 for miR-29 (95% CI 0.736–0.868, *p* < 0.0001) and 0.874 for miR-196 (95% CI 0.810–0.922, *p* < 0.0001), respectively. The optimal sensitivity and specificity to differentiate SVR from NR were (85.8 and 100% at a cutoff value ≤ 689.8) for miR-122, (94.3 and 88.9% at a cutoff value ≤ 71.3) for miR-155, (78 and 77.8% at a cutoff expression value > 0.067) for miR-29, (76.6 and 88.9% at a cutoff value > 0.116) for miR-196, respectively. Comparison of the ROC curve results suggested that the predictive accuracy of serum miR-122 was the most superior; in the order of miR-122 > miR-155 > miR-196 > miR 29 (AUC = 0.973, 0.878, 0.874 and 0.808, respectively) (Fig. 5).

### Laboratory and demographic and data of cases and healthy controls

The current study was performed on (should not it be 141) HCV-patients and 50 healthy controls. The demographic and laboratory data of patients and controls are shown in Table 1. The mean age of the two groups were 45.3 ± 6.9 and 48 ± 7.6 years, respectively. ALT and AST levels were significantly higher in the HCV- patients compared to the healthy controls (*p* value < 0.001*). In contrast, albumin level and PC were significantly lower in HCV- patients compared to controls (*p* value < 0.001*) (Table 1).

**Table 1:** Laboratory and demographic data of cases and controls

	Controls N = 50	Patients N = 150	*p* value
	Mean ± SD	Mean ± SD	
Age	45.2 ± 6.9	48.0 ± 7.6	0.061
Sex			
Males	27 (54%)	72 (48%)	0.462
Females	23 (46%)	78 (52%)	
ALT (U/L)	24.4 ± 2.7	56.0 ± 4.3	< 0.001*
AST (U/L)	21.4 ± 3.1	54.2 ± 4.2	< 0.001*
Albumin (g/Dl)	4.7 ± 0.3	3.9 ± 0.9	< 0.001*
PT	98.4 ± 1.4	89.9 ± 1.8	< 0.001*

*Difference is statistically significant (*p* < 0.05)

### Demographic and clinical data of HCV-patients regarding the response to treatment

Out of 150 HCV- patients, 141 (94%) were responders to (SOF/DAV + RIB) regimen. Only 9 patients were resistant to the treatment (6%). Demographic and clinical data of both SVR and NR (Table 2).

**Table 2:** Demographic and laboratory data of non-responders and sustained virological responders

	NR N = 9	SVR N = 141	*p* value
	Mean ± SD	Mean ± SD	
Age	44.6 ± 10.4	48.2 ± 7.4	0.177
Sex			
Males	5 (55.6%)	67 (47.5%)	0.640
Females	4 (44.4%)	74 (52.5%)	
Platelets (109 /L)	107.6 ± 24.2	120.1 ± 33.4	0.274
ALT (U/L)	51.8 ± 9.7	56.3 ± 4.5	0.751
AST (U/L)	55.6 ± 5.0	54.2 ± 4.2	0.316
Albumin (g/Dl)	3.8 ± 0.9	3.9 ± 0.9	0.886
HCV titer (log EQ/ML)	6.8 ± 1.8	6.2 ± 1.8	0.295
PT	89.8 ± 2.1	89.9 ± 1.8	0.889
AST/ALT ratio	1.1 ± 0.2	1.03 ± 0.17	0.200

### Correlation between miRNAs and liver enzymes and albumin among all studied individuals

Bivariate correlation analysis revealed significant positive correlation between mirRNAs 122, 155 and both ALT and AST levels. It also revealed significant negative correlation between mirRNAs 29, 196 and both ALT and AST levels. In contrast, it revealed significant negative correlation between mirRNAs 122, 155 and serum albumin level and significant positive correlation between mirRNAs 29, 196 and serum albumin level (Table 3).

**Table 3:** Correlation between miRNAs and liver enzymes and albumin among all studied individuals (n = 200)

	MIR-122RQ	MIR155-RQ	MIR29-RQ	MIR196-RQ
ALT, U/L				
Spearman's rho	.497	.532	− .529	− .410
*p* value	< 0.001*	< 0.001*	< 0.001*	< 0.001*
N	200	200	200	200
AST, U/L				
Spearman's rho	.606	.632	− .520	− .407
*p* value	< 0.001*	< 0.001*	< 0.001*	< 0.001*
N	200	200	200	200
Albumin, G/DL				
Spearman's rho	− .234	− .323	.221	.150
*p* value	.001*	< 0.001*	.002*	.034*
N	200	200	200	200

^*^Difference is statistically significant (*p* < 0.05)

### Logistic regression analysis showing association of miRNAs with drug response

Univariate logistic regression analysis revealed that increased mir-196 level is significantly associated with response to DAAs (*p* value = 0.021), while decreased mir-122, 155 levels is associated with response (*p* < 0.001, < 0.001, OR = 0.998, 0.957, CI 0.997–0.999, 0.936–0.978), respectively.

Accordingly, miR-196 level was positive predictor for SVR, while miR-122,155 levels were negative predictors of response. Multivariate logistic regression analysis revealed that mir-196 is the most important marker in predicting response to treatment (*p* value = 0.011) (Table 4).

**Table 4:** Logistic regression analysis showing association of miRNAs with drug response

	B	SE	*p* value	OR	95% CI for OR	
					Lower	Upper
Univariate logistic regression analysis						
Mir-122 RQ	− .002	.001	< 0.001*	.998	.997	.999
Mir-155 RQ	− .044	.011	< 0.001*	.957	.936	.978
Mir-29 RQ	8.788	4.571	.055	6554.555	.842	51,004,936.643
Mir-196 RQ	14.572	6.321	.021*	2,130,709.707	8.871	5.1E
Multivariate logistic regression analysis						
Mir-29 RQ	− 24.082	11.327	.033*	.000	.000	.152
Mir-196 RQ	40.585	16.021	.011*	4.2E	9748.190	1.8E

SE, standard error; OR, odds ratio; CI, confidence interval

*indicates statistical significance (*p* < 0.05)

### Fibrosis categories of the study groups (SVR and NR)

It was observed that there was a significant increase in severity of fibrosis among non-responders as 9 (100%) of them belonged to F2–F3 category (moderate to severe fibrosis). In contrast, most of patients achieving SVR [103 (73.04%)] belonged to F0–F1 category (mild to moderate fibrosis) (*p* < 0.001) (Table 5).

**Table 5:** Fibrosis categories of the study groups (SVR and NR)

	Early fibrosis (F0–F1)	Late fibrosis (F2–F3)
Treatment response		
•Patients who achieved SVR12 (responders)	103 (73.04%)	38 (26.95%)
•Patients who did not achieve SVR12 (non- responders)	0 (0%)	9 (100%)
*p* value	< 0.001*	< 0.001*

### MicroRNAs expression profiles in early and late fibrosis

Non-significant changes of miRNAs expression levels were detected in early and late fibrosis (Table 6).

**Table 6:** MicroRNAs expression profiles in early and late fibrosis

	F0-1 N = 103	F2-3 N = 47	*p* value
MiR-122 RQ			
Median	179.9	118.1	0.947
IQR	35.8–565.6	21.6–950.6	
MiR-155 RQ			
Median	10.13	9.5	0.851
IQR	4.8–33.1	4.4–46.4	
MiR-29 RQ			
Median	0.15	0.2	0.908
IQR	0.08–0.45	0.05–0.5	
MiR-196 RQ			
Median	0.24	0.21	0.644
IQR	0.12–0.58	0.06–0.6	

IQR, interquartile range; RQ, relative quantification

## Discussion

HCV is a global health problem and the major etiological cause of chronic hepatitis and liver disease worldwide. More than 50% of HCV-infected cases develop CHC which can lead to many sequels like fibrosis, cirrhosis, end‐stage liver disease, and HCC [12]. Nowadays, the study of non-invasive diagnostic and prognostic biomarkers of the diseases has become an interesting and rapidly-growing field in clinical research.

Patients who are more or less likely to attain SVR are identified by doctors using predictors of response to therapy as decision-making aids. In the current study, we assessed a number of variables related to HCV infection that affect the prognosis of the illness and how well it responds to therapy. These variables include the amount of expression of a panel of four liver-associated miRNAs in the blood in CHC Egyptian patients getting DAA therapy (SOF/DAV + RIB), as well as in healthy controls.

Regarding HCV replication and infection, microRNAs are thought to be an intriguing area of research. They could also serve as novel targets for the creation of antiviral drugs. Depending on a patient's treatment reaction, miRNA levels vary. In order to reduce unsuccessful therapies, miRNAs can be employed as predictors of patient medication responses before combination therapy [13].

In the current study, A significant overexpression of circulating miR-122 and miR-155 levels was reported in CHC patients compared with healthy controls. These findings are in agreement with other findings that reported increased miR-122 expression [14–16] and increased miR-155 expression [9, 17] in CHC patients. On the other hand, these findings are inconsistent with other data that reported decreased miR-122 expression with advanced fibrosis [18]. This overexpression of miRNAs in serum of HCV‐infected patients may be caused by inflammation and liver cells damage due to viral infection. Inflammation due to viral infection causes damage to hepatocytes resulting in release significant amounts of miRNAs into the circulation, which may explain its high level in serum [19]. miR‐122 facilitates viral RNA replication and plays an important role in the progression of liver disease, therefore it may be considered as a biomarker for HCV infection [20].

On the other hand, the current study has recorded decreased the expression of both miR-29 and miR-196 in CHC patients compared with healthy controls. These findings are consistent with data recorded in previous studies [16, 21] for miR-29 and miR-196 respectively. In contrast to our data El Guendy et al., reported that members of miR-29 and miR-196 families are overexpressed in CHC patients [18]. Downregulation of miR-29 could be explained as HCV might downregulate miR-29 to its advantage. Remarkably, miR-29 overexpression decreased HCV RNA replication. Thus, miR-29 can be deleterious to HCV, providing a possible motivation for the virus to downregulate miR-29 [11]. Roderburg et al. also explained miR-29 down-regulation in liver fibrosis of mice models, where downregulation of miR-29 is mediated by transforming growth factor beta (TGF-β), inflammatory signals lipopolysaccharide (LPS) and nuclear factor kappa B (NF-κB). TGF-β, LPS and NF-κB stimulation leads to decreased miR-29 levels which is associated with increased collagen production leading to fibrosis [22].

Circulating miR-196 is significantly reduced in CHC patients, via decreased release of miR-196 from HCV-infected hepatocytes [21]. Following activation by infection with the hepatitis C virus (HCV), the interferon (IFN) pathway activates miR196 expression. This miRNA either directly inhibits viral replication via a target site on the HCV RNA or indirectly via Bach1 down-regulation and up-regulation of heme oxygenase-1 (HMOX1) [23]. As a result, HCV has evolved to counteract the effect of this antiviral miRNA, however such mechanism remains to be fully investigated. Regulation of miR-196b expression through the (IFN) pathway and inhibition of this pathway by HCV virus particles may explain the lower expression of miR-196 in the cell line after virus transfection and also in the PBMCs of HCV-infected persons [10].

In 2016, the World Health Organization (WHO) updated its guidelines for the screening, care, and treatment of HCV-infected patients to recommend DAA-based regimens instead of IFN-based regimens [24]. Since the publication of the 2016 guidelines, DAA regimens successfully resolve HCV infection in over 85% of treated persons across all six major genotypes. Despite this very high response rate, complete elimination of HCV infection is still not achieved and about 1–15% of HCV-infected patients are still resistant to treatment [25].

In this study, out of 150 patients 141 (94%) have accomplished SVR while 9/150 patients (6%) were non-responders to DAAs treatment. Since treatment is expensive and often has several adverse effects, viral and host factors which impact on the severity of disease and response to treatment becomes more significant [26]. Host miRNAs expressions have been reported to vary with different treatment responses. As a result, miRNAs can be used as biomarkers for predicting response prior therapy, therefore avoiding adverse effects of ineffective treatments, and reducing unnecessary cost. Furthermore, they tend to be new targets for the development of antiviral treatment [27].

The present study reported that the expression levels of miR-122 and miR-155 were significantly higher in non-responders to DAAs than in responders. In contrast, the expression levels of miR-29 and miR-196 were significantly higher in responders to DAAs than in non-responders. ROC analysis showed that all the studied miRNAs could serve as valuable biomarkers for prediction of response to DAAs with AUC 0.973 for miR-122 (95% CI 0.933–0.993, *p* < 0.0001), 0.878 for miR-155 (95% CI 0.815–0.926, *p* = 0.0003), 0.808 for miR-29 (95% CI 0.736–0.868, *p* < 0.0001) and 0.874 for miR-196 (95% CI 0.810–0.922, *p* < 0.0001) respectively. The optimal sensitivity and specificity to differentiate SVR from NR were (85.8 and 100% at a cut-off value ≤ 689.8) for miR-122, (94.3 and 88.9% at a cut-off value ≤ 71.3) for miR-155, (78 and 77.8% at a cut-off expression value > 0.067) for miR-29, (76.6 and 88.9% at a cut-off value > 0.116) for miR-196, respectively. These results suggest that all studied miRNAs could be potential predictive biomarkers differentiating SVR and NR in CHC patients receiving DAAs.

Univariate logistic regression analysis revealed that miR-196 level is positive predictor for SVR, while miR-122,155 levels are negative predictors of response. Multivariate logistic regression analysis revealed that miR-196 is the most important marker in predicting response to treatment (*p* value = 0.011).

Meissner et al., as well investigated differential expression of our four-studied miRNAs before and after SOF/RBV therapy, they reported an increase in expression of miR-122 during treatment-induced HCV clearance (1.4-fold increase, *p* = 0.034), with no change of the other three miRNAs over the course of therapy [28].

Hyrina et al., reported also an increase of miR-122 expression in the serum of NR compared to patients achieving SVR after Interferon-based therapy with first-generation direct-acting antivirals, theses data is consistent with our findings [29]. Although several studies have investigated the role of our four-studied miRNAs as predictors of response to IFN-based regimens [30]. There aren't many studies evaluating their predictive value with DAA-based regimens, according to Meissner and colleagues [28].

To the best of our knowledge, despite the relatively small sample size of the current study, it provided the first clinical evidence of the use of circulating miRNAs (miR; 122, 155, 196, and 29) as diagnostic and predictive biomarkers of CHC in HCV- genotype 4 patients receiving the new DAA regimen (SOF/DAV + RIB). However, more research with a bigger sample size is necessary to thoroughly assess the potential worth of these miRNAs as a useful biomarker.

Circulating miRNAs have become attractive biomarker candidates and are increasingly used in the prevention, diagnosis, prognosis, therapeutic monitoring and even treatment of various human diseases [31].

Bivariate correlation analysis was assessed in the current study between miRNAs expressions and different biochemical Parameters using spearman's correlation test, A significant moderate positive correlation was observed between mirRNAs (miR-122, 155) and liver transaminases levels; [(miR-122): ALT (rho = 0.497); AST (rho = 0.606)], [(miR-155): ALT (rho = 0.532); AST (rho = 0.632)].A significant moderate and weak negative correlation was reported between mirRNAs (miR-29, 196) and liver transaminases levels respectively;[(miR-29): ALT (rho = − 0.529); AST (rho = − 0.520)], [(miR-196): ALT (rho = − 0.410); AST (rho = − 0.407)]. In contrast, bivariate correlation analysis revealed a significant negative weak correlation between mirRNAs 122, 155 and serum albumin level (rho = − 0.234, − 0.323) respectively and a significant positive weak correlation between mirRNAs 29, 196 and serum albumin level (rho = 0.221, 0.150) respectively. The previous observations suggest presence of correlation between the four studied miRNAs and liver function.

Our findings are consistent with some previous studies which reported a positive correlation between miR-122 and liver transaminases levels [19, 32]. Although our data are on contrary with LIU and coworkers’ results which observed no correlation between miR-196 and liver transaminases [21].

We also investigated whether there is any correlation between levels of our four-studied miRNAs with baseline HCV RNA levels; Bivariate correlation analysis revealed positive significant strong correlation between mirRNAs 122, 155 and HCV RNA levels (r = 0.856, *p* < 0.001), (r = 0.798, *p* < 0.001) respectively. A negative significant strong and moderate correlation between mirRNAs 29, 196 and HCV RNA levels was observed (r = − 0.808, *p* < 0.001), (r = − 0.725, *p* < 0.001) respectively.

Our findings agree with a previous study that reported positive correlation between miR-122 and HCV RNA levels [32]. Our results are also in consistent with a previous report showing that overexpression of miR-196b decreases HCV replication by approximately 60% [32]. In contrast to our findings, Motawi et al. [27], reported a negative correlation between miR-122 and HCV RNA levels (r = − 0.278. p = 0.023). Sendi et al. [33]. also reported that miR-122 expression level is negatively correlated with the baseline HCV RNA levels (*r* =  − 0.43, *p* = 0.03), while they found no correlation between miR- 29 and HCV RNA levels. Our data also disagree with LIU et al. [21], who observed no correlation between miR-196 and baseline HCV RNA levels.

## Conclusion

In conclusion, the present study demonstrated that circulating levels of miR-122,155,196 and 29 in serum of HCV-4 patients can be useful biomarkers for CHC prediction, prognosis, treatment outcome predictors in addition to their attractive therapeutic targets. Further studies of larger cohorts are recommended.

## Data Availability

All data generated or analysed during this study are included in this published article.
